# Ponasterone A and F, Ecdysteroids from the Arctic Bryozoan *Alcyonidium gelatinosum*

**DOI:** 10.3390/molecules23061481

**Published:** 2018-06-19

**Authors:** Kine Østnes Hansen, Johan Isaksson, Eirin Glomsaker, Jeanette Hammer Andersen, Espen Hansen

**Affiliations:** 1 Marbio, UiT—The Arctic University of Norway, Breivika, N-9037 Tromsø, Norway; eirin.glomsaker@gmail.com (E.G.); jeanette.h.andersen@uit.no (J.H.A.); espen.hansen@uit.no (E.H.); 2 Department of Chemistry, UiT—The Arctic University of Norway, Breivika, N-9037 Tromsø, Norway; johan.isaksson@uit.no

**Keywords:** marine bryozoan, *Alcyonidium gelatinosum*, ponasterone, ecdysteroids, marine bioprospecting, allelochemicals

## Abstract

A new ecdysteroid, ponasterone F (**1**) and the previously reported compound ponasterone A (**2**) were isolated from specimens of the Arctic marine bryozoan *Alcyonidium gelatinosum* collected at Hopenbanken, off the coast of Edgeøya, Svalbard. The structure of **1** was elucidated, and the structure of **2** confirmed by spectroscopic methods including 1D and 2D NMR and analysis of HR-MS data. The compounds were evaluated for their ability to affect bacterial survival and cell viability, as well as their agonistic activities towards the estrogen receptors α and β. The compounds were not active in these assays. Compound **2** is an arthropod hormone controlling molting and are known to act as an allelochemical when produced by plants. Even though its structure has been previously reported, this is the first time a ponasterone has been isolated from a bryozoan. *A. gelatinosum* produced **1** and **2** in concentrations surpassing those expected of hormonal molecules, indicating their function as defence molecules against molting predators. This work adds to the chemical diversity reported from marine bryozoans and expanded our knowledge of the chemical modifications of the ponasterones.

## 1. Introduction

Ecdysteriods are arthropod steroid hormones controlling molting (ecdysis), development and reproduction through interaction with the ecdysteroid receptors [1]. To date, more than 500 ecdysteroids have been characterized [2]. Compared to human steroidal hormones, ecdysteroids are more hydrophilic due to polyhydroxylation, they have C-27 to C-29 steroidal cores and different shapes due to an A/B-*cis*-ring conjugation [3]. Their structural variations lie in the side chains liked to C-17 of the steroidal D-ring, methylation patterns and conjugation moieties linked through the hydroxyl groups [4,5]. Ecdysteroids are also found in a wide variety of plants [6,7]. Phytoecdysteroids are often present in concentrations surpassing those expected of hormonal molecules [8], and they are widely recognized to serve as anti-feeding agents against insect herbivores [9,10,11].

Ecdysteroids demonstrate several beneficial effects in mammals, including promotion of wound healing, reduction in blood cholesterol and glucose levels and neuroprotective properties [12,13]. In addition, ecdysteroids have the capacity to act as anabolic agents by increasing protein synthesis in skeletal muscle [14]. Ecdysteroids are not prohibited in sports, and a large number of ecdysteroid-based preparations are freely available on the market [15]. Interestingly, this anabolic effect is not associated with the androgenic (masculinization) effects observed for human anabolic-androgenic steroids, like testosterone, as ecdysteroids show no significant binding affinity to the androgen receptor [16]. Humans have no receptor equivalent to the ecdysteroid receptors, and the exact mechanism behind this observed effect is unknown [15]. However, it has been shown that 20-hydroxyecdysone (**3**), the ecdysteroid most commonly administered to humans either naturally through food or as a supplement, mediates its anabolic activity through interaction with the estrogen receptor β [17]. Furthermore, ecdysteroids appear toxicologically benign, with reported LD_50_ > 6 g/kg in mammals [12]. This unique mode-of-action indicates the potential of ecdysteroids as orphan drugs to treat conditions associated with muscle atrophy or injury.

Bryozoans are sessile, marine invertebrates found in benthic ecosystems throughout the world [18]. Bryozoans play relevant roles in nutrient cycles, representing a food source for many marine species, including nudibranchs, and molting invertebrates like arthropods and crustaceans [19]. Their lack of an adaptive immune system and sessile nature has necessitated the production of a wealth of defense molecules [20,21]. Due to the prominent bioactivities of these compounds, many have proven to be important lead structures for pharmaceutical development [22]. Despite the fact that a number of compounds with medical potential have been isolated from bryozoans in recent years, the phylum is relatively under-investigated in this context. In our previous studies that focused on marine bryozoans, a monobrominated tyrosine derivative [23], and five variants of the securamines, as well as their bioactivities have been reported [24].

**Figure 1:**
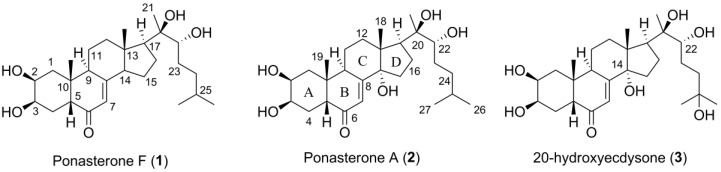
The structures of the herein isolated compounds ponasterone F (**1**) and A (**2**) and 20-hydroxyecdysone (**3**), a similar ecdysteroid reported in literature.

During the course of our investigation towards the discovery of bioactive secondary metabolites from Arctic marine bryozoans, *Alcyonidium gelatinosum* was extracted and fractionated. Preliminary bioactivity experiments showed that one of the fractions significantly inhibited the viability of a human melanomae cancer cell line. Further evaluation of the chemical constituents of the organic extract led to the isolation of a new ponasterone, ponasterone F (**1**), along with ponasterone A (**2**) (Figure 1), which has been isolated previously from several sources, including the leaves of the terrestrial plant *Podocarpus nakaii* [25]. As part of this study, the ponasterones were isolated from a bryozoan for the first time. Herein, we report the isolation, structure elucidation and bioactivity profiling of the two compounds, including their estrogen receptor agonistic activities. Molting in bryozoans is an almost unrecognized phenomenon. The free-living bryozoan species *Cupuladria doma* has been shown to molt frontal and basal membranes under conditions of heavy fouling colonies [26]. It has also been described for *Alcyonidium sanguineum*, a species closely related to *A. gelatinosum* (same family). We therefore hypothesize the role of the ponasterones to be part of a chemical defense system for *A. gelatinosum*.

## 2. Results and Discussion

### 2.1. Biomass Collection, Extraction and Fractionation

Alcyonidium gelatinosum was collected with a triangular scrape from Hopenbanken, Svalbard. Freeze-dried *A. gelatinosum* (wet weight 5700.1 g) was extracted with water. The resulting pellet was dried (dry weight: 498.9 g) and extracted twice in MeOH:CH_2_Cl_2_ (1:1). After filtration, the solvent was evaporated under reduced pressure, yielding the organic extract (27.43 g). The organic extract was fractionated into eight fractions using RP Flash chromatography.

### 2.2. Viability Screening of the Flash Fractions

The flash fractions were assayed for activity against the human melanoma cancer cell line A2058. Fraction 5 (eluting at 100% MeOH) exhibited significant cytotoxic activity against the cell line: 1% and 17% cell survival at 50 and 25 µg/mL, respectively. The remaining fractions were inactive.

### 2.3. Dereplication of the Active Fraction

**Figure 2:**
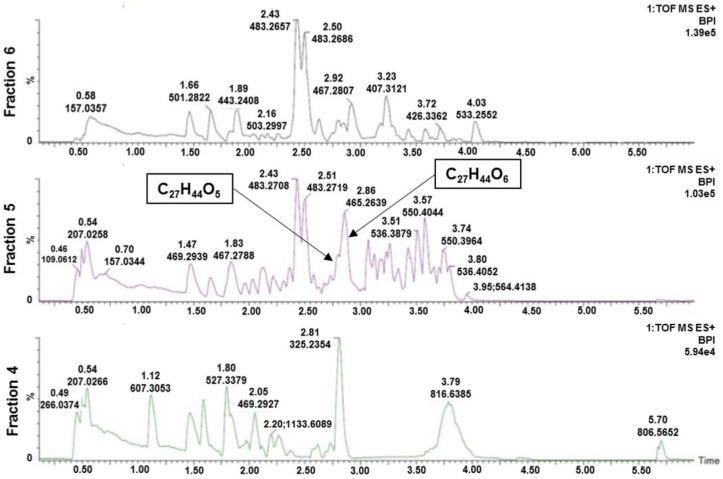
Chromatograms of the active fraction 5, and the inactive fractions 4 and 6, analyzed using UHPLC-HR-MS with positive electrospray. Two compounds, with elemental compositions C_27_H_44_O_5_ (**1**) and C_27_H_44_O_6_ (**2**), were found in significantly higher amounts in the active fraction compared to the inactive fractions.

In an attempt to identify compound(s) responsible for the observed activity, flash fraction 5 of *A. gelatinosum* was analysed using UHPLC-HR-MS. The inactive fractions 4 and 6 were also analysed to enable comparison between the content of the active and inactive fractions. Two compounds, with elemental compositions C_27_H_44_O_5_ (**1**) and C_27_H_44_O_6_ (**2**) were found in substantial amounts in the active fraction (Figure 2). The two compounds were not present in fraction 4, and in significantly lower amounts in fraction 6. The two elemental compositions gave no hits in searches against the MarinLit database (http://pubs.rsc.org/marinlit/). The two compounds were therefore selected for isolation.

### 2.4. Isolation of **1** and **2**

Compound **1** and **2** were isolated from the organic extract using mass guided semi-preparative HPLC. Stepwise purification using RP HPLC columns with fluoro-phenyl and C_18_ packing material led to the isolation of one novel ponasterone analog, ponasterone F (**1**) (5.47 mg) along with the previously reported ponasterone A (**2**) (1.25 mg).

### 2.5. Structure Elucidation of Compound **1**

Compound **1** had the molecular formula C_27_H_44_O_5_ as established from the [M + H]^+^ peak in HR-ToF-MS analysis, which indicates that it lacked one hydroxy group compared to **2** (C_27_H_44_O_6_). Close inspection of ^1^H and ^13^C-NMR data (Table 1) of **1** confirmed the presence of 27 carbons and 44 hydrogens. 2D NMR data recorded for **1** established that the compound had a C27-steroidal structure, and furthermore revealed close structural similarity to that of **2**, which also was isolated and submitted to NMR analysis as part of this study (Table S1).

**Figure 3:**
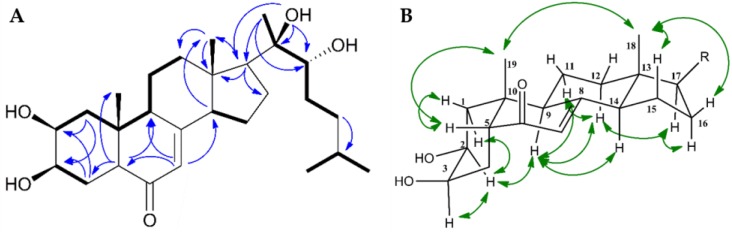
(**A**) Key COSY (bold) and HMBC (blue arrows) correlations for ponasterone F (**1**); (**B**): Selected 2D ROESY (green arrows) NMR correlations for **1**.

Compared to **2**, the ^1^H-NMR spectrum of **1** showed an additional proton signal at 2.13 ppm bound to C-14 (shifed from 83.10 to 54.98 ppm relative to **2**). In addition, the 14-OH signal, recorded for **2** at 5.62 ppm, was absent. The remaining NMR data corresponded well to **2**. Further evaluation of HMBC, HSQC, COSY and ROESY spectra of **1** were consistent with **1** and **2** having identical steroidal rings, both in terms of structure and configuration (Figure 3).

In the side chain of the steroid, the preseance of a tertiary alcohol on C-20 was confirmed by key long-range ^n^J_CH_ couplings from C-17, C-22 and C-20 to OH-20 and a secondary alcohol on C-22 was confirmed by a ^3^J_HH_ coupling between H-22 and OH-22. The cis junction of rings A/B was proven by Hα-9/Hα-2 and H_3_-19/Hβ-5 correlations in the ROESY spectra of **1**. Moreover, the H_3_-18/H_3_-19, H_3_-18/Hβ-16, H_3_-18/Hβ-15, Hα-12/Hα-17, and H14α/H9α ROE correlations confirmed the trans junctions of rings C/D (Figure 3B). The configuration at C-22, as well as the configuration and individual assignment of the geminal Me-26 and Me-27 groups were ambiguous due to their mutual HMBC correlations and rotational freedom of the side chain. The reported configuration of the side chain as well as the absolute configuration of the steroid is based on analogy to data reported in literature [17,27,28,29].

### 2.6. Estrogen Receptor Agonist Activities of **1** and **2**

The ecdysteroid 20-hydroxyecdysone (**3**) (Figure 1) has potent agonistic effect towards human estrogen receptors α and β with EC_50_ values of 26 and 13 nM, respectively, causing skeletal muscle hypertrophy in rats [17]. We therefore tested the structurally closely related **1** and **2** for agonistic activity towards the two receptors in a LanthaScreen ^TM^ TR-FRET based assay conducted by ThermoFisher Scientific (Madison, WI, USA). The compounds were not found to have agonistic activity towards the two receptors up to concentrations of 10 µM. It has been shown that hydroxylation of C-14 and C-22 is important for the binding behavior of ecdysteroids [30]. Compounds **3** and **2** are hydroxylated at C-14 and C-22, while **1** lacks the C-14 methoxy. However, the presence or absence of specific substituents is not sufficient for strong discrimination of bioactivity. It is thus likely that a combination of factors, like spatial orientation of functional groups and their influence on the overall tree-dimensional structure, affect the observed difference in agonistic activity.

### 2.7. Antiproliferative and Antibacterial Properties of **1** and **2**

As the ponasterone containing fraction of the organic extract of *A. gelatinosum* was found to be active against the human melanoma cell line A2058, and the isolated compounds **1** and **2** were assayed for cytotoxic properties against A2058 and the non-malignant human fibroblasts MRC-5. Compounds **1** and **2** did not affect the survival of these cell lines at concentrations up to 215 and 223 µM, respectively. This is in line with previous studies, finding the lethal dose of ecdysteroids in mammals to be 6 g/kg [12,15]. Further isolation of compounds from the organic extract of *A. gelatinosum* is thus necessary to identify the compound(s) responsible for the bioactivity observed for flash fraction 5. Furthermore, **1** and **2** showed no activity against the human pathogenic bacterial strains *Staphylococcus aureus*, *Enterococcus faecalis*, *Escherichia coli*, *Pseudomonas aeruginosa*, and *Streptococcus agalactiae* at the highest assayed concentration (**1**: 215 µM, **2**: 223 µM).

### 2.8. Probable Natural Functions of **1** and **2** in A. gelatinosum

Out of all known ecdysteroids, **2** is known to have strongest affinity for the ecdysone receptor [31]. Compound **2** is well known to be involved in molting in insects and a few animals [32,33]. When phytoecdysteroids are produced by plants, it is widely recognized that their benefit to the producing organism is to repel insect herbivores [34]. The same is true for some marine organisms, including the sea spider Pycnogonum litorale [35]. The silk worm Bombay mori demonstrates physiological responses to dietary level of **2** as low as 0.03 mg **2**/kg fresh weight of ingested plant [36]. The concentrations of **1** and **2** in *A. gelatinosum* were 13.25 and 3.02 mg/kg wet animal weight, respectively. These high levels of ecdysteroids would argue against their function as hormones, but rather indicates that they have an antifeeding effect making *A. gelatinosum* unpalatable to molting predators like crustaceans.

## 3. Materials and Methods

### 3.1. General Experimental Procedures

Optical rotations were measured on an AA-10R automatic polarimeter (Optical activity LTD, Ramsey, UK) in MeOH. NMR spectra were acquired in DMSO-*d*_6_ on a Bruker Avance III HD spectrometer (BioSpin, Fallanden, Switzerland) operating at 600 MHz for protons, equipped with an inverse TCI cryo probe enhanced for ^1^H, ^13^C and ^2^H. All NMR spectra were acquired at 298 K, in 3 mm solvent matched Shigemi tubes using standard pulse programs for Proton, Carbon, HSQC, HMBC, DQCOSY, and ROESY with gradient selection and adiabatic versions where applicable. ^1^H/^13^C chemical shifts were referenced to the residual solvent peak (DMSO-*d*_6_: δH = 2.50, δC = 39.51). HRESIMS were performed using a Waters LCT Premier Time-of-Flight MS with an Acquity UPLC (Waters, Milford, MA, USA), using MS grade solvents and a Waters Acquity UPLC BEH (1.7 µm, 2.1 × 100 mm) column. Compound isolation was performed using a preparative-HPLC-MS system consisting of a 600 HPLC Pump, a 2996 Photodiode Array UV detector, a 3100 Mass detector, and a 2767 sample manager (Waters). The following columns were used: Atlantis Prep C_18_ (10 µm, 10 × 250 mm), Xselect CSH Prep Fluoro-phenyl (5 µm, 10 × 250 mm), Xselect CSH Phenyl-Hexyl Prep (5 µm, 10 × 250 mm), and Xterra Prep RP C_18_ (10 µm, 10 × 300 mm). (All from Waters). Flash chromatography was carried out using a Biotage HPFC SP4 system equipped with a Biotage SNAP column filled with 8 g Diaion HP-20SS (Supelco Analytical, Charlottesville, NA, USA). All solvents used for extraction and fractionation were of HPLC grade and Milli-Q H_2_O was used.

### 3.2. Biological Material

Specimens of the bryozoan *Alcyonidium gelatinosum* (class Gymnolaemata, order Ctenostomatida, family Alcyonidiidae) were collected with a triangular bottom scrape in May 2014 at Hopenbanken, Svalbard (75.5168 N, 23.9793 E, at 72 m depth). The organism was identified by Robert A. Johansen of the Norwegian national biobank (Marbank), and a voucher specimen (ref. M14055) was deposited in Marbank, Tromsø, Norway. The specimen was stored at −23 °C in the dark until processed.

### 3.3. Extraction and Fractionation

Freeze-dried *A. gelatinosum* (wet weight 5700.1 g) was diced and extracted twice with water at 4 °C in the dark. After supernatant removal, the remaining pellet was freeze-dried (dry weight: 498.9 g) and extracted twice in 50:50 MeOH:CH_2_Cl_2_ (*v*:*v*). After filtration, the solvent was pooled and evaporated under reduced pressure, and a dark solid residue (27.43 g) was obtained. An aliquot of the extract (1.503 g) was then fractioned using flash chromatography on a prepacked column filled with Dainon HP-20SS resin (8 g). The following step gradient was applied: MeOH/H_2_O to MeOH in five steps (5:95, 25:75, 50:50, 75:25, 100:0) followed by MeOH:acetone to acetone in two steps (50:50, 0:100) with a flow rate of 12 mL/min. A total of eight fractions were collected (72 mL each) and dried under reduced pressure.

### 3.4. Cytotoxicity Screening (MTS Assay) of the Flash Fractions

The flash fractions were assayed at concentrations of 50 µg/mL for activity against the human melanoma cell line A2058 as previously described [24].

### 3.5. Dereplication of the Active Fraction

Flash fractions 4, 5, and 6 (0.04 µg in 3 µL) were injected onto the UHPLC column. Compound separation was achieved using water and acetonitrile (both with 0.1% formic acid) as the mobile phase. The following gradient was employed: 20–100% acetonitrile over 5 min followed by 100% acetonitrile for 1 min at 0.55 mL/min. The resulting chromatograms were manually inspected to identify compound exclusively present, or present in significantly higher concentrations, in the active fraction.

### 3.6. Isolation of **1** and **2**

The organic extract of *A. gelatinosum* (1.99 g) was partitioned between 150 mL hexane and 150 mL 90% MeOH three times. The pooled MeOH fractions were dried under reduced pressure and redissolved in 31 mL MeOH. Aliquots of the sample were repeatedly injected onto a XSelect CSH phenyl-hexyl column and eluted with a mobile phase consisting of solvents A (MQ-water and 0.1% formic acid) and B (acetonitrile and 0.1% formic acid), delivered in a gradient mode at 6 mL/min, starting from B at 10% to 70% over 20 min, and then B at 100% for 1 min. The compounds were collected with retention times of **1**: 14.3 and **2**: 12.2 min, respectively. Final separation of the compounds from sample impurities was achieved by an extensive series of isolation steps. Aliquots of **1**, dried and dissolved in MeOH, was injected onto a fluoro-phenyl column. Compound **1** eluted after 13.8 min (gradient: 10–52% B over 14 min). The pooled and dried fractions were then finally dissolved in MeOH and injected onto an XTerra C_18_ column from which **1** eluted after 15.8 min (gradient: 10–61% B over 17 min). Aliquots of **2** isolated from the organic *A. gelatinosum* extract were dissolved in MeOH and injected onto a fluoro-phenyl column from which **2** eluted after 11.5 min (gradient: 10–46% B over 12 min). The pooled and dried fractions were then finally dissolved in MeOH and injected onto an Atlantis C_18_ column. Compound **2** eluted after 13.6 min (gradient: 10–52% B over 14 min). In the end, the two compounds were isolated in amounts that enabled structure determination by NMR analysis: **1** (5.47 mg) and **2** (1.25 mg).

Compound **1**: white powder; [α]${}_{D}^{20}$ 15 ± 0.02 (c 0.2 MeOH); ^1^H and ^13^C-NMR data in Table 1; HRESIMS *m*/*z* 449.2682 [M + H]^+^ (calcd. for C_27_H_45_O_5_, 449.2691).

### 3.7. Agonistic Activity of **1** and **2** against Estrogen Receptors Alpha and Beta

The agonistic activity of **1** and **2** against the estrogen receptors α and β were assayed in LanthaScreenTM time-resolved fluorescence resonance energy transfer (TR-FRET) assays found in the SelectScreenTM biochemical nuclear receptor profiling service at ThermoFisher SCIENTIFIC (Madison, WI, USA). Compounds **1** and **2** were assayed in 10-point titrations (three-fold serial dilution), with 10 µM as the highest assay concentration. The agonist assays protocol and data analyzed were conducted as described by the service provider [37].

### 3.8. Antiproliferative and Antibacterial Properties of **1** and **2**

The antiproliferative activities of **1** and **2** were tested against the human melanoma cell line A2058 and the non-malignant lung fibroblasts MRC-5 as previously described [24]. The antibacterial properties of **1** and **2** against the human pathogenic bacterial strains *Staphylococcus aureus*, *Enterococcus faecalis*, *Escherichia coli*, *Pseudomonas aeruginosa*, and *Streptococcus agalactiae* were assayed as previously described [38]. The compounds were assayed in six-point titration curves. The highest assayed concentration of **1** was 215 µM and the highest assayed concentration of **2** was 223 µM in all antiproliferative and antibacterial assays.

## Figures and Tables

**Table 1:** ^1^H- and ^13^C-NMR data for ponasterone F (**1**) in DMSO-d_6_.

Ponasterone F (1)			
Position	*δ*_C_, Type	*δ*_H_ (*J* in Hz)	*δ*_OH_ (*J* in Hz)
1	36.6, CH_2_	1.25, dd, 13.3, 11.9/1.58, dd, 13.3, 4.3	
2	66.7, CH	3.64, dt, 11.7, 3.3	
3	66.7, CH	3.73, d, 3.8	4.35
4	31.8, CH_2_	1.49, m ^1,2^	4.37
5	50.1, CH	2.19, dd, 11.9, 5.3	
6	201.9, C		
7	120.7, CH	5.45, s	
8	164.9, C		
9	37.5, CH	2.59, t, 7.7	
10	37.3, C		
11	21.4, CH_2_	1.75, ddt, 13.4, 6.6, 3.1/1.59, m ^1,2^	
12	38.7, CH_2_	2.15, m ^1,2^/1.49, m ^1,2^	
13	45.0, C		
14	55.0, CH	2.13, m ^1,2^	
15	22.01, CH_2_	1.55, m ^1,2^/1.44, m ^1,2^	
16	21.3, CH_2_	1.89, m ^2^/1.51, m ^1,2^	
17	54.4, CH	1.66, t, 9.4	
18	14.0, CH_3_	0.71, s	
19	24.1, CH_3_	0.83, s	
20	75.4, C		3.62
21	20.8, CH_3_	1.08, s	
22	75.5, CH	3.12, dd, 10.0, 1.7	4.36
23	29.0, CH_2_	1.38, m ^1,2^/1.09, m ^1,2^	
24	36.1, CH_2_	1.37, m ^1,2^/1.13, m ^1,2^	
25	27.4, CH	1.50, m ^1,2^	
26	23.0, CH_3_	0.86, d, 6.6	
27	22.3, CH_3_	0.85, d, 6.6	

^1^ Overlapping peaks; ^2^ δ_H_ extracted from HSQC.

## Supplementary Materials

The following are available online. Figures S1–S10: Supplementary data (^1^H, ^13^C, HSQC, HMBC and ROESY NMR spectral data of ponasterone F (**1**) and ponasterone A (**2**); Table S1: containing ^1^H and ^13^C-NMR data of **2**).
