# Supplementary material for: Ponasterone A and F, Ecdysteroids from the Arctic Bryozoan Alcyonidium gelatinosum
Source: Molecules. 2018 Jun 19;23(6):1481. doi: 10.3390/molecules23061481 (PMC6100090; doi:10.3390/molecules23061481)
Supplement: Supplementary file 1 [file molecules-23-01481-s001.pdf]

Supplementary Materials

Ponasterone A and F, ecdysteroids from the Arctic bryozoan *Alcyonidium gelatinosum*

Kine Ø. Hansen[1,*], Johan Isaksson[2], Eirin Glomsaker[1], Jeanette H. Andersen[1] and Espen Hansen[1]

Affiliation

[1] Marbio, UiT – The Arctic University of Norway, Breivika, N-9037, Tromsø, Norway;

[2] Department of Chemistry, UiT – The Arctic University of Norway, Breivika, N-9037, Tromsø, Norway;

***** Correspondence: kine.o.hanssen@uit.no; Tel.: +47-77649272

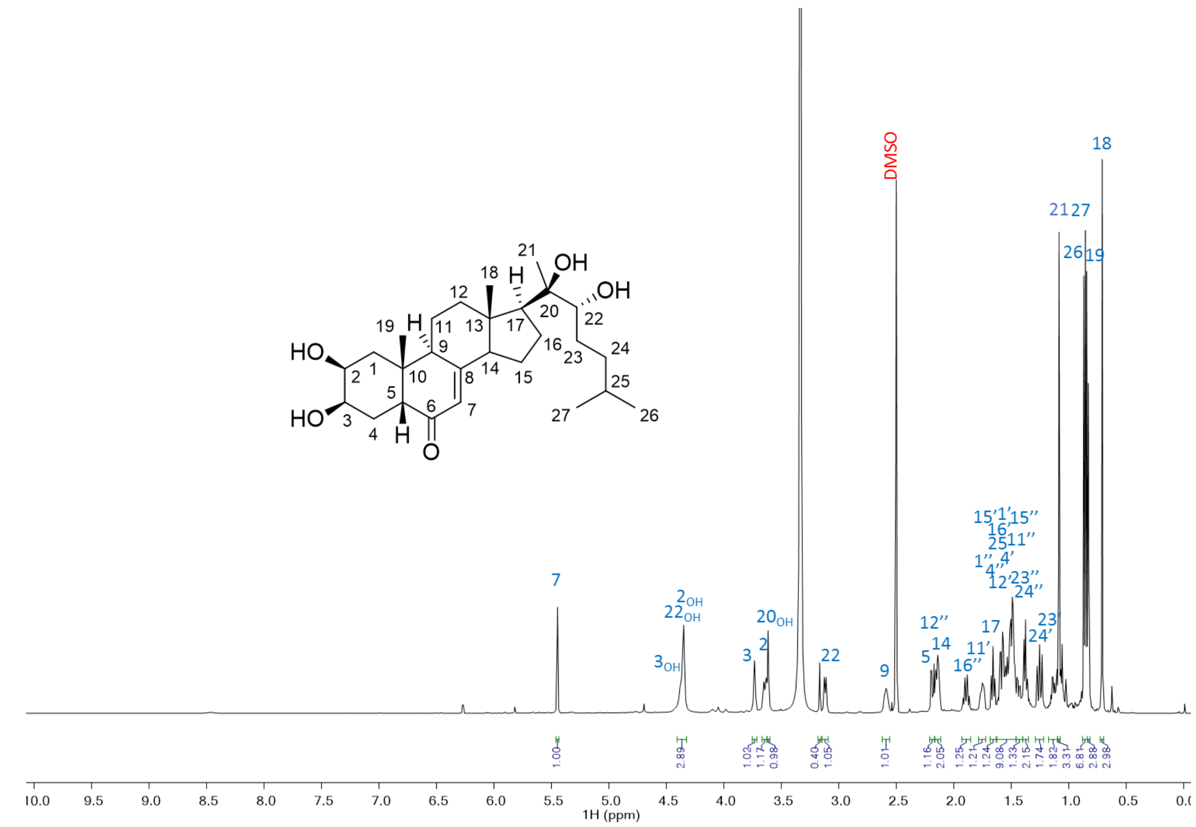

Figure S1. ^{1}H NMR (600 MHz, DMSO-d_6) spectrum of ponasterone F (**1**)

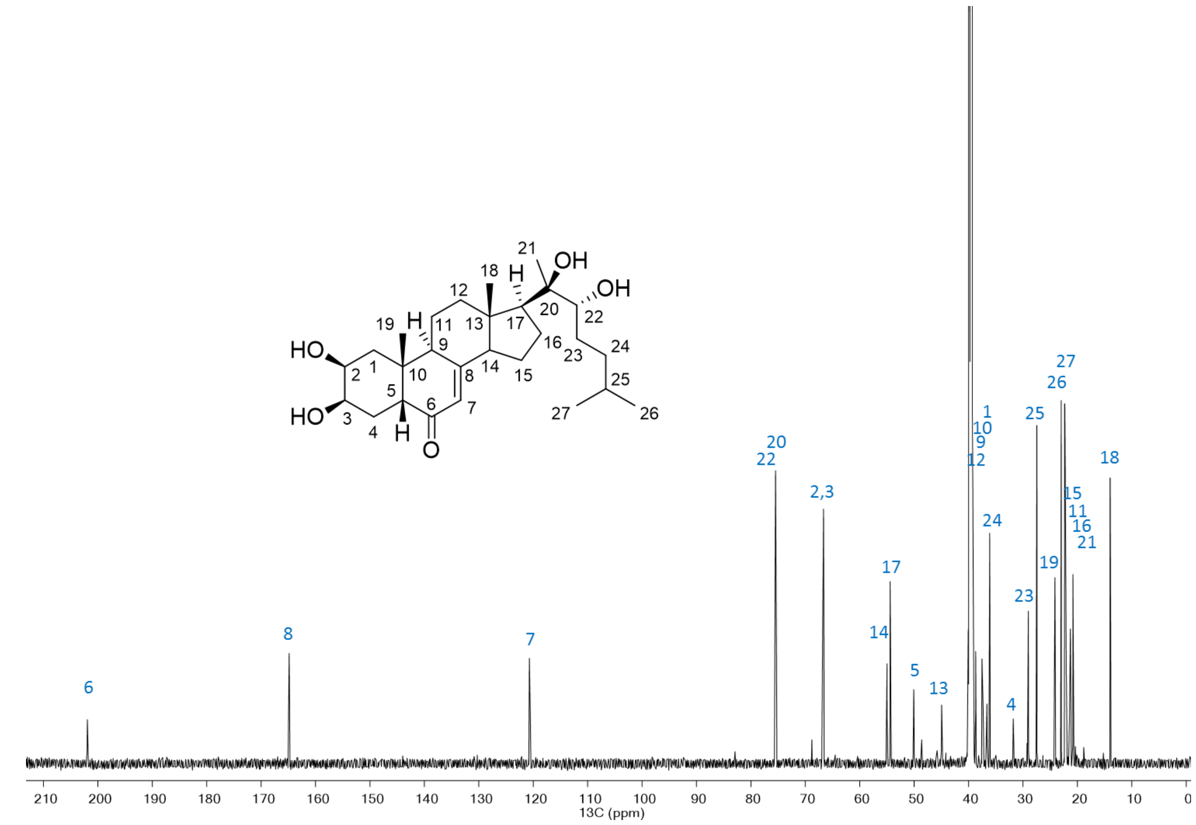

Figure S2. ^{13}C (151 MHz, DMSO-d_6) spectrum of ponasterone F (**1**)

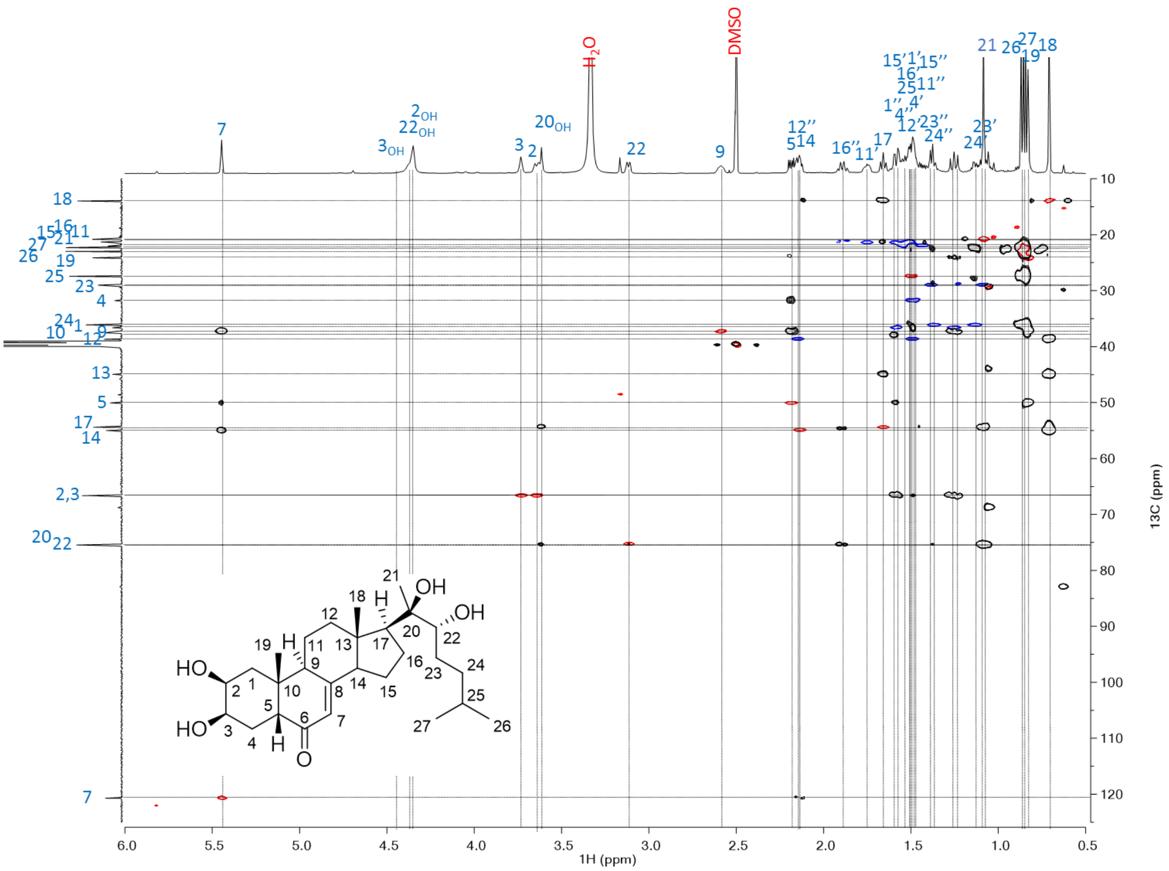

Figure S3. HSQC + HMBC (600 MHz, DMSO-d_6) spectrum of ponasterone F (**1**)

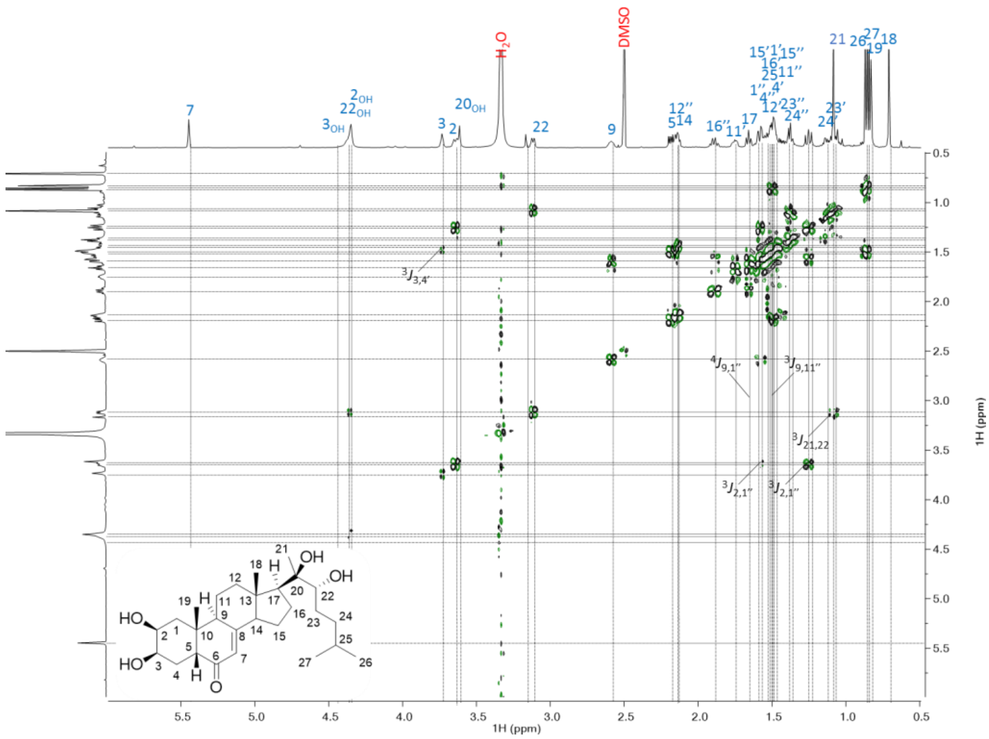

Figure S4. COSY (600 MHz, DMSO-d_6) spectrum of ponasterone F (**1**)

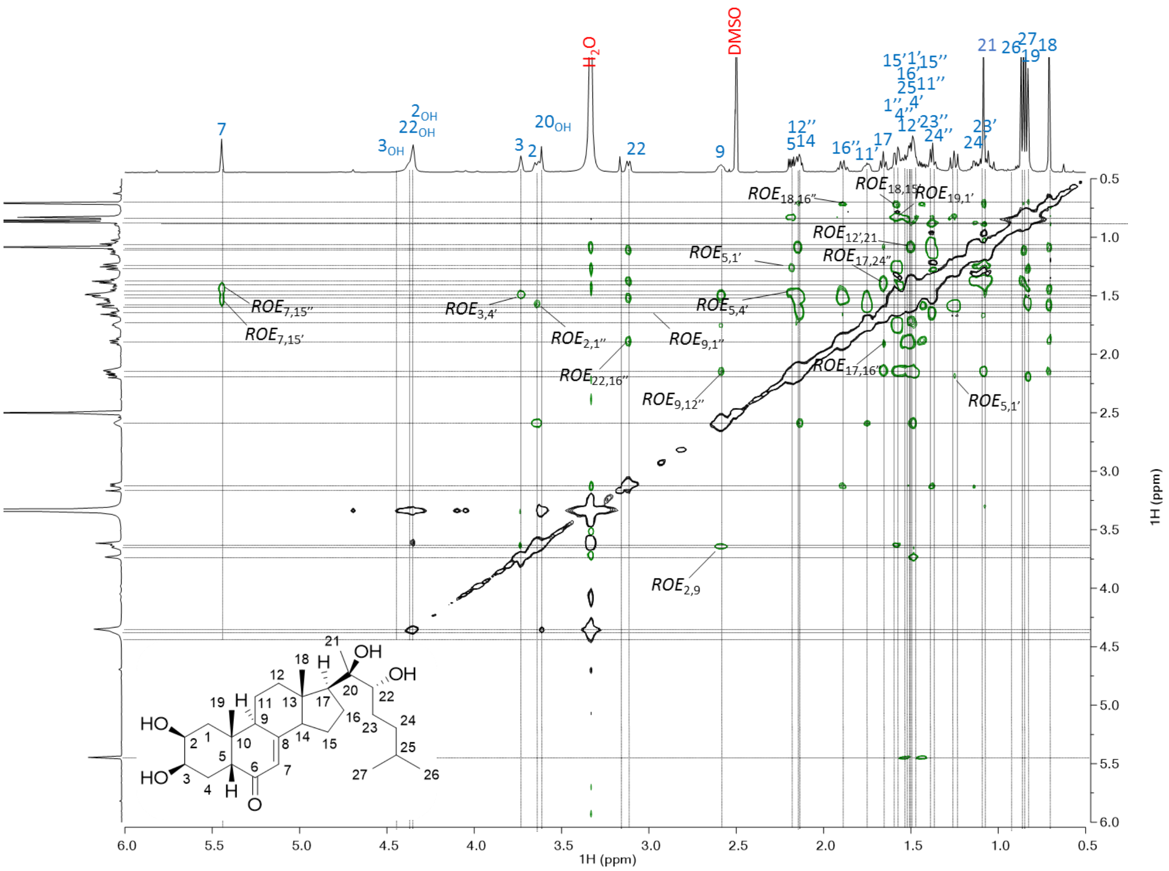

Figure S5. ROESY (600 MHz, DMSO-d_6) spectrum of ponasterone F (**1**)

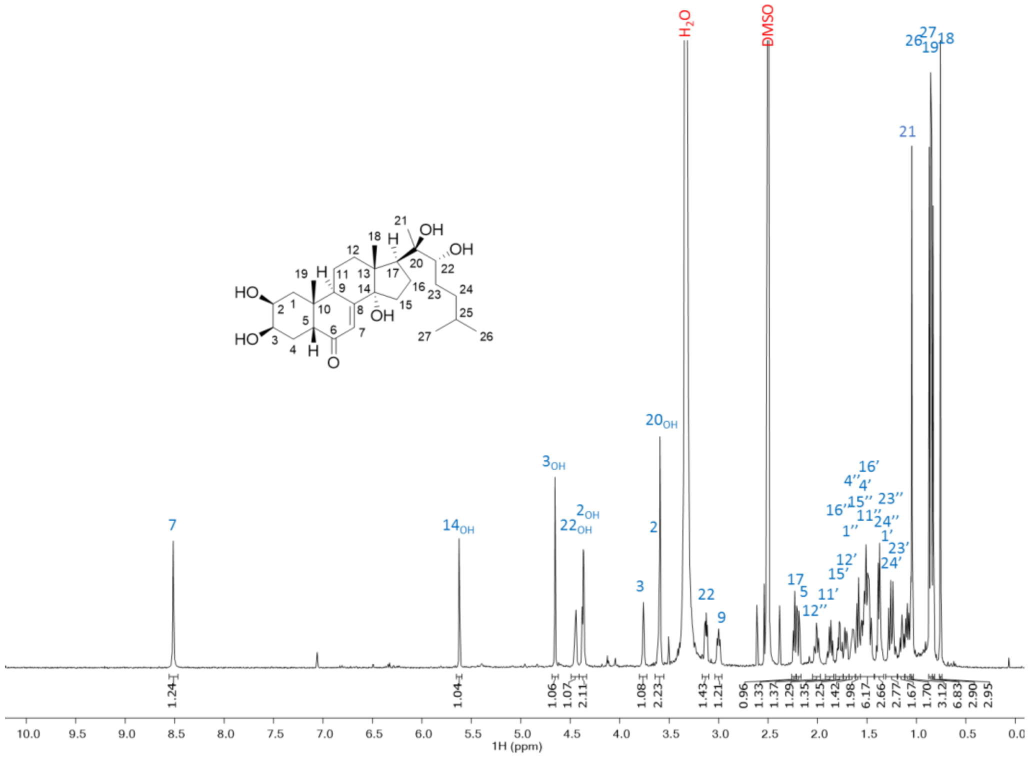

Figure S6. ^{1}H NMR (600 MHz, DMSO-d_6) spectrum of ponasterone A (**2**)

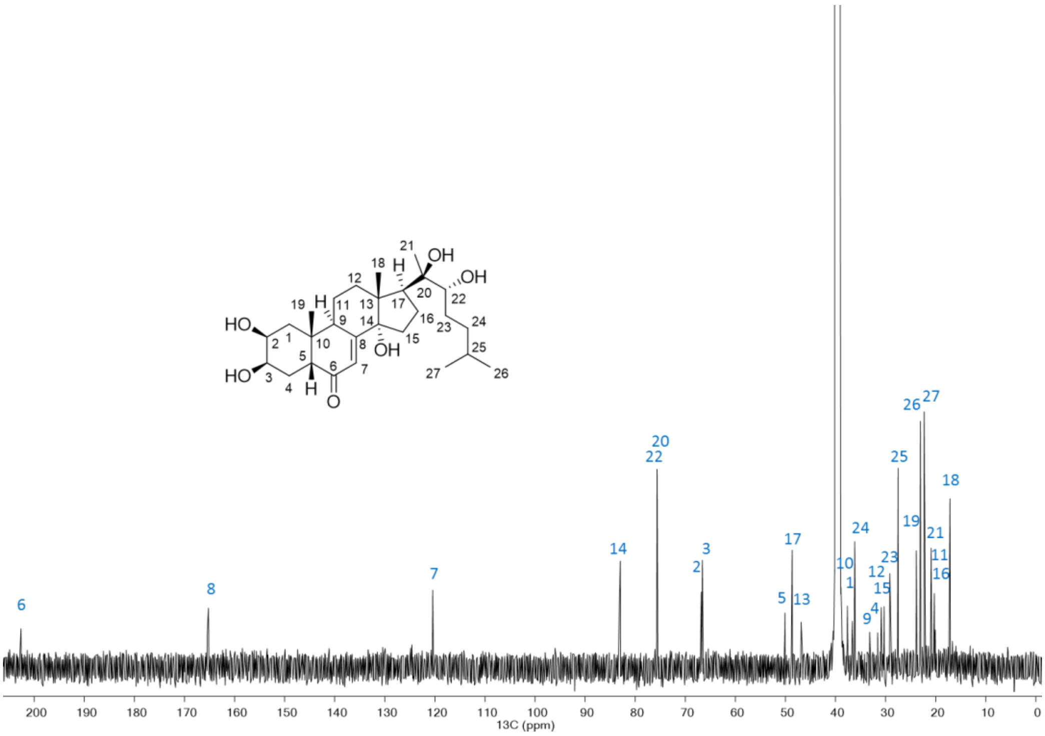

Figure S7. ^{13}C (151 MHz, DMSO-d_6) spectrum of ponasterone A (**2**)

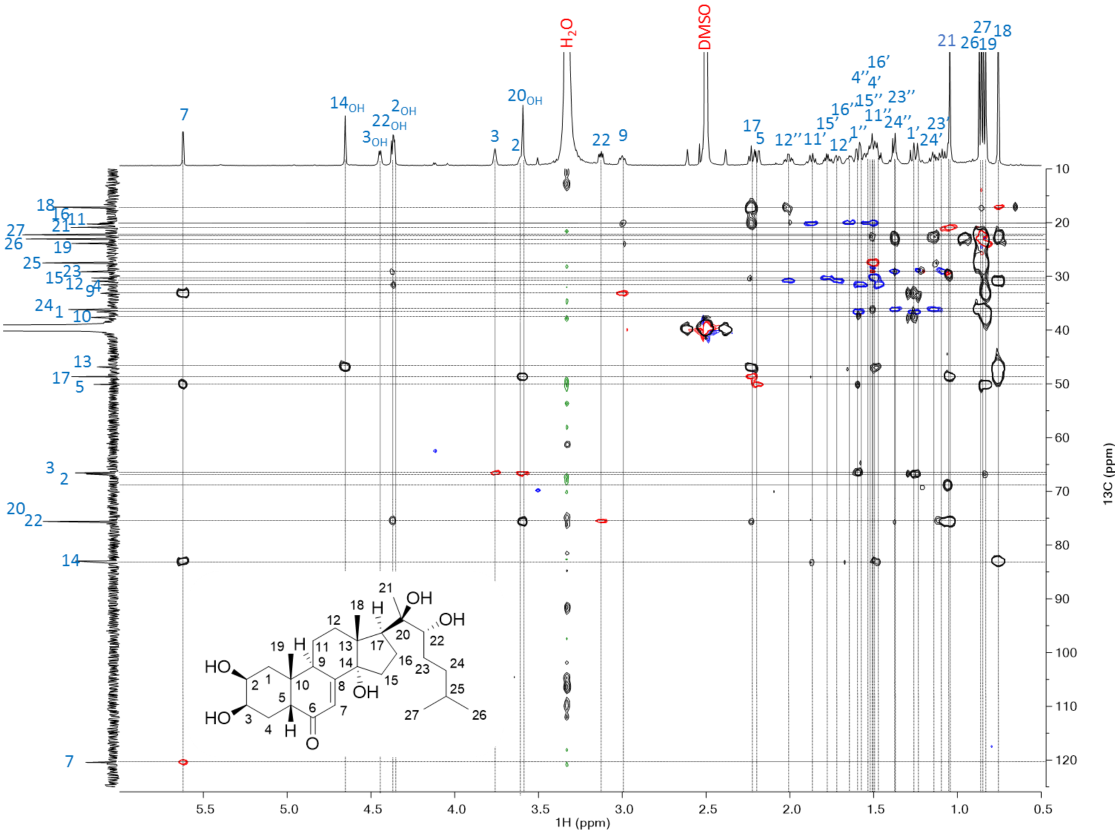

Figure S8. HSQC + HMBC (600 MHz, DMSO-d_6) spectrum of ponasterone A (**2**)

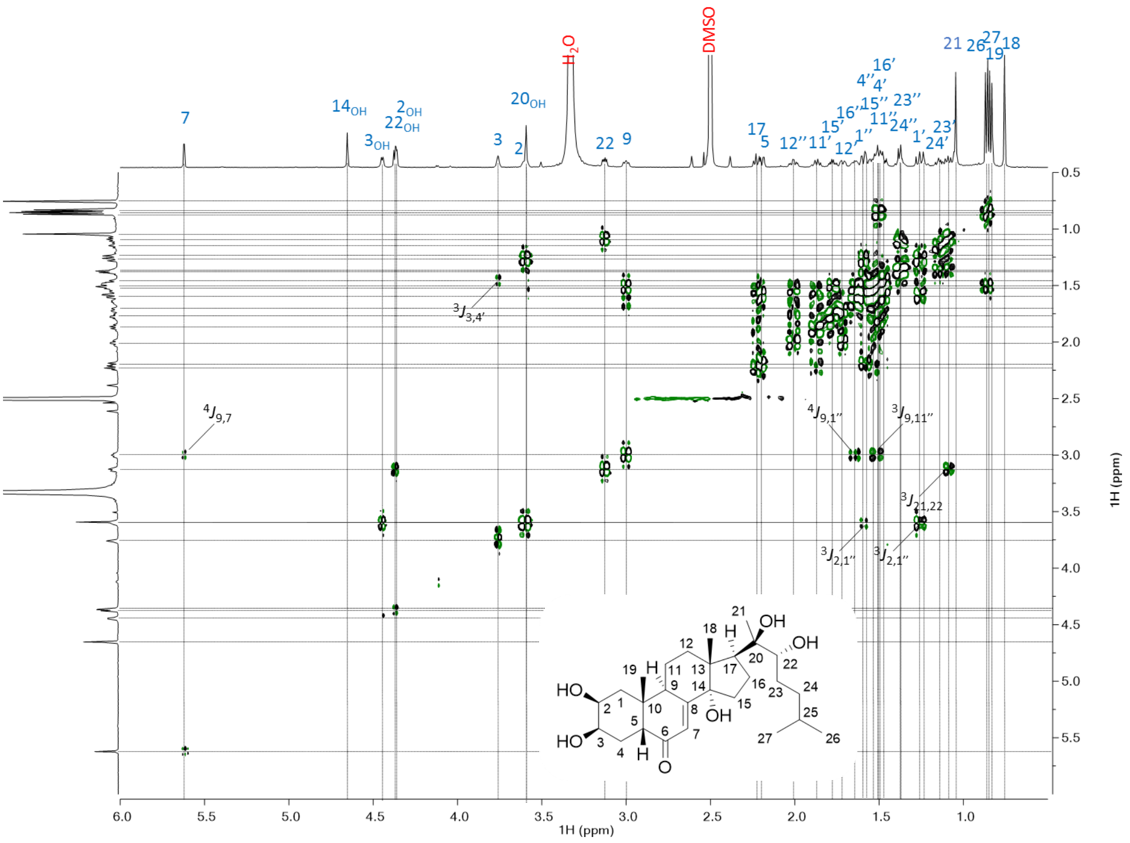

Figure S9. COSY (600 MHz, DMSO-d_6) spectrum of ponasterone A (**2**)

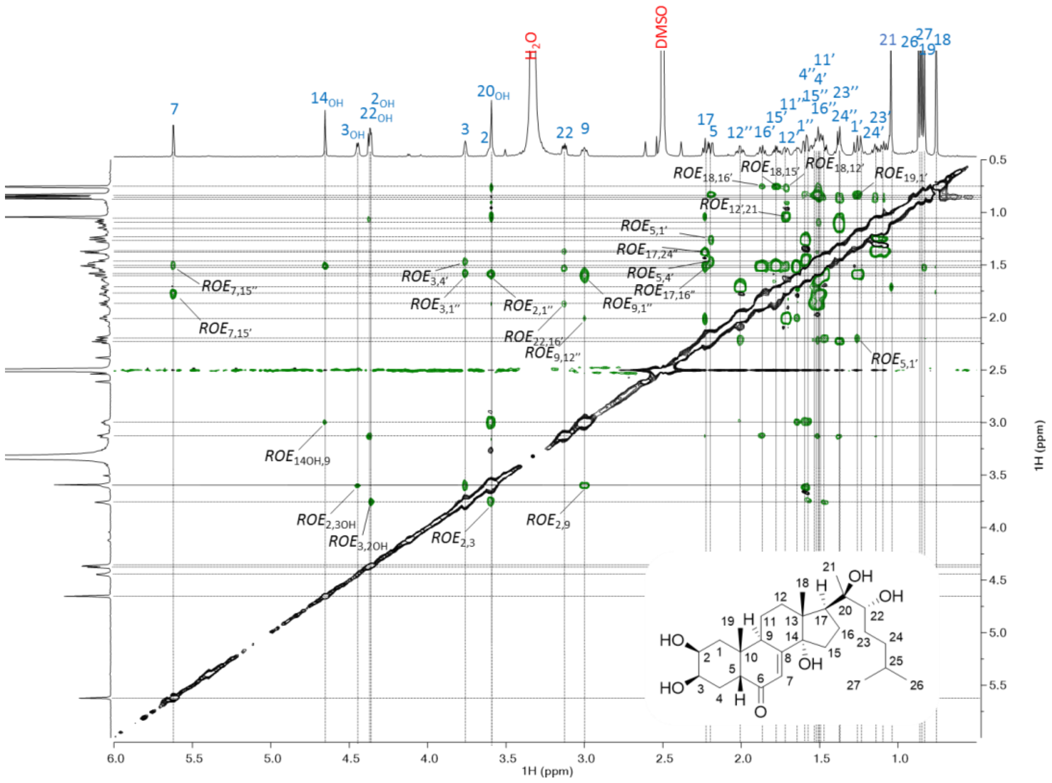

Figure S10. ROESY (600 MHz, DMSO-d_6) spectrum of ponasterone A (**2**)

Table S1. ^{1}H- and ^{13}C-NMR data for ponasterone A (**2**) in DMSO-d_6

position	δ_C, type	δ_H (*J* in Hz)	δ_{OH} (*J* in Hz)
1	36.6, CH	1.26, t, 12.7/1.59, dd, 13.3, 3.7	
2	66.8, CH	3.60, m[b]	4.37, dd, 6.7, 4.2
3	66.6, CH	3.76, s[b]	4.65, s
4	31.6, CH_2	1.57, m[o]/1.46, m[o]	
5	50.1, CH	2.20, dd, 13.2, 4.2	
6	202.7, C		
7	120.4, CH	8.51, s	
8	165.2, C		
9	33.2, CH	3.00, ddd, 11.1, 7.4, 1.7	
10	37.6, C		
11	20.3, CH_2	1.87, q, 10.5, 9.7/	
12	30.9, CH_2	1.72, dd, 12.5, 2.9/2.01, td, 12.9, 4.7	
13	46.9, C		
14	83.0, C		5.62, d, 2.5
15	30.3, CH_2	1.78, td, 12.1, 11.4, 5.6/1.50, m[o]	
16	20.1, CH_2	1.53, m[o]/1.65, dd, 12.5, 6.0	
17	48.7, CH	2.23, t, 9.5	
18	17.1, CH_3	0.76, s	
19	23.9, CH_3	0.83, s	
20	75.6, C		3.59, s
21	20.9, CH_3	1.05, s	
22	75.6, CH	3.13, dd, 9.8, 4.9	4.45, d, 5.8
23	29.1, CH_2	1.08, m/1.37, m[o]	
24	36.1, CH_2	1.14, m/1.37, m[o]	
25	27.5, CH	1.50, m[o]	
26	23.0, CH_3	0.86, d, 6.6	
27	22.3, CH_3	0.85, d, 6.6	

[b]broad peak [o]overlapping

*Ponasterone A (**2**):* light yellow powder; $[\alpha]^{20}_{D}$ 29 ±0.02 (*c* 0.07 MeOH); ^{1}H and ^{13}C NMR data in Table S1; HRESIMS *m/z* 465.2636 $[M + H]^+$ (calcd for $C_{27}H_{44}O_6$, 465.2641).
